# Antimicrobial Susceptibility Trends Observed in Urinary Pathogens Obtained From New York State

**DOI:** 10.1093/ofid/ofy297

**Published:** 2018-11-16

**Authors:** Elliot L Rank, Thomas Lodise, Lisa Avery, Eve Bankert, Erica Dobson, Ghinwa Dumyati, Stephen Hassett, Marina Keller, Matthew Pearsall, Teresa Lubowski, Joseph J Carreno

**Affiliations:** 1 Microbiology, Quest Diagnostics, Teterboro, New Jersey; 2 Pharmacy Practice, Albany College of Pharmacy and Health Sciences, Albany, New York; 3 Pharmacy Practice, Wegmans School of Pharmacy/St. John Fisher College, Rochester, New York; 4 Island Peer Review Organization, Albany, New York; 5 Pharmacy, University of Rochester Medical Center, Rochester, New York; 6 Infectious Diseases Division, University of Rochester, Rochester, New York; 7 Emergency Medicine, Albany Med EmUrgent Care, Albany, New York; 8 Infectious Disease, Orange Regional Medical Center, Middletown, New York; 9 Pharmacy, Glens Falls Hospital, Glens Falls, New York

**Keywords:** antibiogram, antimicrobial resistance, New York State, urinary tract infection

## Abstract

International guidelines recommend using local susceptibility data to direct empiric therapy for acute uncomplicated cystitis. We evaluated outpatient urinary isolate susceptibility trends in New York State. Nitrofurantoin had the lowest resistance prevalence whereas trimethoprim-sulfamethoxazole and fluoroquinolones had higher prevalences. This study highlights the need for local outpatient antimicrobial stewardship programs.

Acute uncomplicated cystitis is a prevalent outpatient condition [1, 2]. It is estimated that there are approximately 6–8 million annual visits to a physician or clinic by patients for treatment of an acute uncomplicated cystitis event [3, 4]. Treatment guidelines for acute uncomplicated cystitis in premenopausal, nonpregnant women are well defined in international guidelines [2]. In these international guidelines, empiric treatment recommendations are provided, but these empiric recommendations are accompanied by the caveat that empiric antibiotic selection should be guided by local organism susceptibility data and patient-specific risk factors.

Although treatment selection for acute uncomplicated cystitis was straightforward in the past, management of these conditions has been complicated by reports of rising antibiotic resistance rates for several key urinary pathogens. It is important to note that much of the data that describe rising rates of resistance for uncomplicated cystitis pathogens were derived from hospitalized inpatients with urinary tract infections. Data that describe outpatient resistance rates are consistent with published inpatient reports but are limited [5]. It is also unclear if resistance rates among acute uncomplicated cystitis pathogens are applicable to all age groups, and to both men and women. Given these gaps in the literature, this study sought to describe the prevalence and resistance patterns of urinary pathogens in New York State in the outpatient setting. The intent was to use these data to help inform treatment decisions for patients who present with uncomplicated cystitis in the outpatient setting and to assess the appropriateness of empiric treatment recommendations found in national guidelines for New York State.

## METHODS

A retrospective analysis was conducted on all urine cultures received from outpatient settings (defined as physician offices or outpatient clinics), with antimicrobial susceptibility testing performed from January 1, 2016, to December 31, 2016, at a major clinical microbiology reference laboratory (Quest Diagnostics Laboratory, Teterboro, NJ). As antimicrobial testing is not routinely recommended for *Streptococcus agalactiae* and *Staphylococcus saprophyticus,* these organisms were not included in the analysis. Data from 17 New York State counties were included in the overall sample (Table 1). Urine cultures demonstrating 1 or 2 bacterial isolates at >10^5^ colony-forming units/mL were included (only the first reported isolate from dual infections was included). In accordance with standards for summarizing antimicrobial susceptibilities for antibiograms, each species required a minimum of 30 isolates for inclusion in antimicrobial susceptibility estimates.

**Table 1.** Summary of Urinary Isolate Antibiotic Susceptibility From New York State

Microorganism	N^a^	Ampicillin	Ceftazidime	Cefazolin	Ciprofloxacin	Nitrofurantoin	Pip-Tazo	Tobramycin	TMP-SMX	Tetracycline
Overall^b^										
Gram-negative										
* Citrobacter diversus*	933		100		99	90	99	100	99	
* Citrobacter freundii*	316		93		95	95	94	98	85	
* Enterobacter aerogenes*	785		93		99	30	97	100	99	
* Enterobacter cloacae*	404		91		94	44		96	86	
* Escherichia coli*	50 900	53	99	95	78	97	97	90	73	
* Klebsiella pneumoniae*	7734		100	98	95	42	95	96	90	
* Proteus mirabilis*	3389	79	99	91	90		100	95	87	
* Providencia rettgeri*	43		93		85		95			
* Pseudomonas aeruginosa*	1051		94		76		92	95		
* Serratia marcescens*	264		100		98			88	97	
* Stenotrophomonas maltophila*	53								100	
Gram-positive										
Methicillin-sensitive *Staphylococcus aureus*	825				85	98			98	89
Methicillin-resistant *Staphylococcus aureus*	326				25	97			96	83
Vancomycin-sensitive *Enterococcus* spp.	9281	100				99				
Vancomycin-resistant *Enterococcus faecium*	35	94				97				
Females <18 y^c^										
Gram-negative										
* Escherichia coli*	3872	41	99	97	91	98		91	74	
* Klebsiella pneumoniae*	281		100	97	97	46		98	92	
* Proteus mirabilis*	264	80	100	89	96			94	88	
* Pseudomonas aeruginosa*	71		97		100			100		
Gram-positive										
* Enterococcus* spp.	441	100				100				
Methicillin-sensitive *Staphylococcus aureus*	61				93	98			97	92
Females 18–64 y^d^										
Gram-negative										
* Citrobacter diversus*	498		100		100	92		100	99	
* Citrobacter freundii*	91		99		97	96		98	88	
* Enterobacter aerogenes*	433		96		99	16		100	100	
* Enterobacter cloacae*	126		91		98	46		98	86	
* Escherichia coli*	29 180	57	99	97	84	98		92	75	
* Klebsiella pneumoniae*	3642		100	97	86	38		87	80	
* Proteus mirabilis*	1486	84	100	94	96			97	91	
* Pseudomonas aeruginosa*	109		94		84			95		
* Serratia marcescens*	67		100		100			88	97	
Gram-positive										
Methicillin-sensitive *Staphylococcus aureus*	430				92	98			99	87
Methicillin-resistant *Staphylococcus aureus*	105				54	94			98	65
Vancomycin-sensitive *Enterococcus* spp.	4425	100				99				
Females >64 y^e^										
Gram-negative										
* Citrobacter diversus*	190		98		98	86		99	97	
* Citrobacter freundii*	140		91		93	97		100	88	
* Enterobacter aerogenes*	154		88		100	55		100	99	
* Enterobacter cloacae*	123		92		98	47		96	88	
* Escherichia coli*	13 156	53	99	92	67	96		88	71	
* Klebsiella pneumoniae*	2781				46	21		43	34	
* Proteus mirabilis*	978	76	99	88	84			95	83	
* Pseudomonas aeruginosa*	312		97		85			98		
* Serratia marcescens*	37		100		97			86	100	
Gram-positive										
Methicillin-sensitive *Staphylococcus aureus*	106				74	98			100	91
Methicillin-resistant *Staphylococcus aureus*	79				11	99			94	94
Vancomycin-sensitive *Enterococcus* spp.	1843	100				98				
Males <18 y^f^										
Gram-negative										
* Escherichia coli*	126	52	99	94	91	98		92	72	
* Klebsiella pneumoniae*	21		100	97	99	46		100	91	
* Proteus mirabilis*	108	70	100	86	96			93	85	
Gram-positive										
Vancomycin-sensitive *Enterococcus* spp.	124	99				100				
Males 18–64 y^g^										
Gram-negative										
* Citrobacter diversus*	96		100		100	95		100	100	
* Enterobacter aerogenes*	83		87		98	30		100	94	
* Enterobacter cloacae*	34		97		94	38		97	88	
* Escherichia coli*	1862	48	99	96	70	97		85	69	
* Klebsiella pneumoniae*	318		100	98	99	41		99	94	
* Proteus mirabilis*	157	72	99	85	83			93	80	
* Pseudomonas aeruginosa*	139		93		65			93		
* Serratia marcescens*	54		100		98			89	96	
Gram-positive										
Methicillin-sensitive *Staphylococcus aureus*	68				84	100			97	93
Vancomycin-sensitive *Enterococcus* spp.	990	100				99				
Males >64 y^h^										
Gram-negative										
* Citrobacter diversus*	104		100		97	88		99	97	
* Citrobacter freundii*	49		86		96	90		94	72	
* Enterobacter aerogenes*	93		89		97	23		100	99	
* Enterobacter cloacae*	91		89		85	37		93	81	
* Escherichia coli*	2704	43	98	92	51	94		82	64	
* Klebsiella pneumoniae*	691		100	98	95	45		97	92	
* Proteus mirabilis*	396	74	98	87	75			92	82	
* Pseudomonas aeruginosa*	405		92		66			93		
* Serratia marcescens*	90		100		97			90	97	
Gram-positive										
Methicillin-sensitive *Staphylococcus aureus*	145				70	99			98	94
Methicillin-resistant *Staphylococcus aureus*	110				7	99			96	92
Vancomycin-sensitive *Enterococcus* spp.	1458	100				99				

The 17 New York counties are Albany, Bronx, Dutchess, Fulton, Greene, Kings, Nassau, New York, Orange, Putnam, Queens, Richmond, Rockland, Suffolk, Sullivan, Ulster, and Westchester.

Abbreviations: Pip-Tazo, piperacillin-tazobactam; TMP-SMX, trimethoprim/sulfamethoxazole.

^a^N = total number of antimicrobial testing results.

^b^Data not shown for 1739 coagulase-negative staphylococci.

Organisms not reported due to <30 isolates in the reporting group:

^c^Females <18 years: *Citrobacter diversus* (29 isolates), *Citrobacter freundii* (11), *Enterobacter aerogenes* (19), *Enterobacter cloacae* (27), methicillin-resistant *S. aureus* (5), *Providencia rettgeri* (1), *Serratia marcescens* (7), *Staphylococcus haemolyticus* (9), *Staphylococcus hominis* spp. *hominis* (4), *Staphylococcus ludgunensis* (1), *Staphylococcus simulans* (25), *Stenotrophomonas maltophila* (2), vancomycin-resistant *Enterococcus faecium* (0).

^d^Females 18–64 years: *Providencia rettgeri* (5), *Providencia stuartii* (4), *Staphylococcus hominis* spp. *hominis* (9), *Staphylococcus ludgunensis* (29), *Stenotrophomonas maltophila* (8), vancomycin-resistant *Enterococcus faecium* (3).

^e^Females >64 years: *Providencia rettgeri* (16), *Providencia stuartii* (13), *Staphylococcus hominis* spp. *hominis* (10), *Staphylococcus ludgunensis* (25), *Staphylococcus simulans* (28), *Stenotrophomonas maltophila* (10), vancomycin-resistant *Enterococcus faecium* (11).

^f^Males <18 years: *Citrobacter diversus* (16), *Citrobacter freundii* (4), *Enterobacter aerogenes* (3), *Enterobacter cloacae* (3), *Klebsiella pneumoniae* (21), methicillin-resistant *S. aureus* (0), methicillin-sensitive *S. aureus* (15), *Providencia rettgeri* (1), *Pseudomonas aeruginosa* (15), *Serratia marcescens* (9), *Staphylococcus haemolyticus* (9), *Staphylococcus hominis* spp. *hominis* (3), *Staphylococcus ludgunensis* (0), *Staphylococcus simulans* (24), *Stenotrophomonas maltophila* (1), vancomycin-resistant *Enterococcus faecium* (0).

^g^Males 18–64 years: *Citrobacter freundii* (21), methicillin-resistant *S. aureus* (27), *Providencia rettgeri* (2), *Providencia stuartii* (2), *Staphylococcus hominis* spp. *hominis* (11), *Staphylococcus ludgunensis* (7), *Staphylococcus simulans* (1), *Stenotrophomonas maltophila* (8), vancomycin-resistant *Enterococcus faecium* (2).

^h^Males >64 years: *Providencia rettgeri* (18), *Providencia stuartii* (13), *Staphylococcus hominis* spp. *hominis* (28), *Staphylococcus ludgunensis* (25), *Staphylococcus simulans* (16), *Stenotrophomonas maltophila* (24), vancomycin-resistant *Enterococcus faecium* (19).

Urine pathogens were isolated from bi-plates of Trypticase Soy Agar with 5% Sheep Blood (TSA II) and MacConkey II Agar, followed by identification and automated drug susceptibility testing (Vitek-2). Antimicrobials tested included ampicillin, ceftazidime, cefazolin, ciprofloxacin, nitrofurantoin, piperacillin-tazobactam, trimethoprim-sulfamethoxazole, tetracycline, oxacillin, and vancomycin, as appropriate. Minimum inhibitory concentration (MIC) susceptibility interpretations were derived from CLSI M100 S-25 [6]. Antibiotic sensitivity percentages are reported as number of susceptible isolates divided by number of isolates tested. Susceptibility was reported overall and by sex and age (children: ≤17 years; adults: 18–64 years; and older adults: ≥65 years). Chi-square tests or Fisher exact tests were used to compare the overall prevalence of bacterial resistance within and between agents tested and between age and sex groups.

## RESULTS

A total of 78 078 urine culture susceptibility reports were included (Table 1). The majority of the urine cultures were obtained from female patients. The most frequently recovered isolates were *Escherichia coli*, 65.1%; *Enterococcus* spp., 11.9%; and *Klebsiella pneumoniae*, 10.0%. Among isolates recovered from females, the distribution was consistent with the overall study population. In men, the prevalence of *E. coli* was lower (40.3 %) relative to the overall population, whereas the prevalence of *Enterococcus* was higher (22.1%). In children, *E. coli* was the most prevalent isolate (73.7%), followed by *Enterococcus* (10.4%) and *Proteus* species (6.8%). The distribution of pathogens in adult (age 18–64 years) patients was similar to the overall population: *E. coli* (68.5%), *Enterococcus* (11.2%), and *K. pneumoniae* (8.7%). In older adults, the prevalence of *E. coli* was lower (58.7%), whereas the prevalence rates of *Enterococcus* and *Klebsiella* were higher (12.2% and 12.8%, respectively).

Of all the isolates tested for nitrofurantoin sensitivity (n = 73 191), 90.4% were susceptible. Nitrofurantoin resistance was more commonly noted in males as compared with females (10.6% vs 9.1%, *P* < .001) and older adults as compared with all other ages combined (12.3% vs 8.1%, *P* < .001). High overall rates of susceptibility were reported for isolates tested for cefazolin sensitivity (90.4%). However, due to the high prevalence of *Enterococcus* spp. (which is intrinsically resistant to cefazolin), cefazolin only has activity against 68.2% of the isolates. Only 77.2% of all isolates tested (n = 67711) were trimethoprim-sulfamethoxazole susceptible. Resistance to trimethoprim-sulfamethoxazole was more prevalent in men than women (26.3% vs 22.7%, *P* < .001) and in older adults than all other age groups combined (25.1% vs 22.1%, *P* < .001). Of the isolates tested for ciprofloxacin sensitivity (n = 68 709), 80.2% were susceptible. Resistance to ciprofloxacin was more frequent in males than females (35.0% vs 17.3%, *P* < .001) and in older adults than in all other ages combined (30. % vs 14.0%, *P* < .001). Additional data on pathogen-specific sensitivity overall and by sex and age groups are presented in Table 1. County-specific data are provided in the Supplementary Data.

## DISCUSSION

The current Infectious Disease Society of America (IDSA) guidelines for acute uncomplicated cystitis recommend nitrofurantoin, trimethoprim-sulfamethoxazole, or fosfomycin as firstline agents for treatment [2]. Overall, we found that bacterial isolates in the outpatient setting in New York State were 90% sensitive *in vitro* to nitrofurantoin. The high probability of *in vitro* activity with nitrofurantoin is likely due to the high prevalence and susceptibility rates for *E. coli*. Although the IDSA guidelines do not define a resistance prevalence threshold for assessing the appropriateness of nitrofurantoin for empiric use in treating acute uncomplicated cystitis, if we apply the trimethoprim-sulfamethoxazole threshold (20%, per the IDSA guidelines), our data support the utilization of nitrofurantoin in the New York State outpatient setting for this condition. In contrast, trimethoprim-sulfamethoxazole appears to have more limited utility as an empiric treatment regimen for acute uncomplicated cystitis as the overall prevalence of trimethoprim-sulfamethoxazole resistance exceeded 20%. Unfortunately, fosfomycin susceptibility data were unavailable in this data set as testing was not routinely performed. Given the limited activity of trimethoprim-sulfamethoxazole and ciprofloxacin for acute uncomplicated cystitis, it would be prudent for laboratories to consider testing of fosfomycin in urinary isolates, especially for *Escherichia coli* and *Enterococcus faecalis*. In species other than *Escherichia coli* and *Enteroccocus faecalis*, the utility and validity of susceptibility results for fosfomycin have yet to be determined [6]. Importantly, recent data show that 1 day of fosfomycin is inferior to 5 days of nitrofurantoin for acute uncomplicated cystitis [7]. As such, fosfomycin should be used with caution when treating acute uncomplicated cystitis.

Susceptibility results from this study were also unfavorable to the empiric use of fluoroquinolones and β-lactam antibiotics. The overall prevalence of ciprofloxacin resistance was 19.8%, exceeding the IDSA-recommended 10% resistance threshold for empiric use. In light of the relatively high prevalence of fluoroquinolone resistance as well as growing concerns about fluoroquinolone-associated disability, our study supports the recent Food and Drug Administration recommendation to avoid empiric fluoroquinolone use unless no other alternative agents are available [7]. We also examined cefazolin as a surrogate for cephalexin susceptibility. When only Gram-negative bacterial species were isolated, cefazolin appeared to be an appropriate empiric agent. However, the utility of cefazolin as an empiric agent is less than favorable due to the high prevalence of *Enterococcus*, an organism that is intrinsically resistant to cefazolin. Given that β-lactam medications require longer duration of treatment as compared with other therapies and are associated with lower efficacy [8], our data suggest that β-lactam drugs should be considered as empiric agents only when potential benefits outweigh risks.

These data also indicate that the overall antimicrobial susceptibility percentages of antimicrobials are significantly lower for individuals in the ≥65 age group compared with all other ages combined and among males of all ages compared with females. These findings highlight the need to not rely exclusively on overall susceptibility results and to consider sex, age, and prior urine culture results when selecting an agent for a given patient. As susceptibility rates varied by age and sex, subsequent adjustment of therapy based on individualized culture and susceptibility reports should be performed in an effort to promote use of narrow-spectrum antibiotics where possible.

There are several caveats to be considered with respect to these findings. We did not include coagulase-negative staphylococci in this analysis due to its questionable pathogenicity. However, our conclusions would be affected minimally by the inclusion of coagulase-negative staphylococci because coagulase-negative staphylococci only accounted for 2% of isolates. Another potential limitation of this study is lack of data on clinical presentation of the patients. We did not determine whether these were symptomatic urinary tract infections or asymptomatic bacteriuria. Last, this study may overrepresent antimicrobial resistance rates as practitioners may only send urine cultures for patients with recurrent infections or treatment failure.

Although these data are from New York State, there are several generalizable aspects of this study. First, this project highlights the framework for creating a regional antibiogram. Regional antibiograms are important tools that could be used by health departments and other regional authorities to help influence prescribing and/or track antimicrobial resistance for key pathogens. Second, New York State (especially Kings, Queens, New York, Bronx, and Richmond counties, the 5 New York City counties) is unique in that it has an incredibly high population density [9]. Along with antimicrobial utilization, population density has been shown to be associated with antimicrobial resistance prevalence [10]. Hence, it stands to reason that New York State can be thought of as a regional bellwether for antimicrobial resistance.

In summary, we conducted a 1-year retrospective analysis of outpatient urine isolates collected from patients in New York State. Data indicated that nitrofurantoin has retained activity against many of the urinary pathogens since the publication of the 2011 IDSA guidelines and has a high prevalence of *in vitro* activity. In contrast, we found that the overall prevalence of resistance in bacterial urinary isolates exceeded the predefined IDSA empiric therapy thresholds of 20% for trimethoprim-sulfamethoxazole and 10% for fluoroquinolones, with resistance rates being highest in patients ≥65 years. These data highlight the need in outpatient settings for (1) urinary culture specimen submission, (2) antimicrobial stewardship efforts, and (3) assembly of local susceptibility data to guide empiric therapy in acute uncomplicated cystitis. Finally, these data also support the desperate need for new oral antimicrobials to treat acute uncomplicated cystitis in the outpatient setting.

## Supplementary Data

Supplementary materials are available at *Open Forum Infectious Diseases* online. Consisting of data provided by the authors to benefit the reader, the posted materials are not copyedited and are the sole responsibility of the authors, so questions or comments should be addressed to the corresponding author.

Supplemental Table 1

Supplemental Table 2

Supplemental Figure 1

Supplemental Figure 2

Supplemental Figure 3
