# Supplementary material for: Antimicrobial Susceptibility Trends Observed in Urinary Pathogens Obtained From New York State
Source: Open Forum Infect Dis. 2018 Nov 16;5(11):ofy297. doi: 10.1093/ofid/ofy297 (PMC6284462; doi:10.1093/ofid/ofy297)
Supplement: Supplemental Table 1 [file ofy297_suppl_supplemental_table_1.docx]

Supplemental Table 1 – County Antibiograms

|  | n | Ampicillin | Ceftazidime | Cefazolin | Ciprofloxacin | Nitrofurantoin | Levofloxacin | Tobramycin | TMP/SMX | Tetracycline |
| --- | --- | --- | --- | --- | --- | --- | --- | --- | --- | --- |
| Albany |  |  |  |  |  |  |  |  |  |  |
| *Escherichia coli* | 311 | 60 | 99 | 97 | 90 | 96 | 93 | 93 | 81 |  |
| *Klebsiella pneumoniae* | 32 |  | 100 | 100 | 100 | 47 | 100 | 100 | 100 |  |
| Bronx |  |  |  |  |  |  |  |  |  |  |
| *Citrobacter diversus* | 62 |  | 100 |  | 98 | 92 | 98 | 100 | 98 |  |
| *Enterobacter aerogenes* | 49 |  | 86 |  | 94 | 10 | 94 | 100 | 98 |  |
| Enterococcus species | 413 | 100 |  |  |  | 99 |  |  |  |  |
| *Escherichia coli* | 2132 |  | 92 | 88 | 97 | 97 | 73 | 89 | 68 |  |
| *Proteus mirabilis* | 176 | 80 | 99 | 83 | 90 |  | 88 | 97 | 85 |  |
| *Pseudomonas aeruginosa* | 53 |  | 94 |  | 70 |  | 66 | 94 |  |  |
| *Staphylococcus aureus MRSA* | 43 |  |  |  | 84 | 100 | 86 |  | 98 | 90 |
| Dutchess |  |  |  |  |  |  |  |  |  |  |
| Enterococcus Species | 112 | 97 |  |  |  | 96 |  |  |  |  |
| *Escherichia coli* | 827 | 60 | 95 | 92 | 83 | 98 | 84 | 93 | 81 |  |
| *Klebsiella pneumoniae* | 95 |  | 100 | 100 | 100 | 37 | 99 | 100 | 96 |  |
| *Proteus mirabilis* | 41 | 78 | 100 | 93 | 93 |  | 93 | 100 | 85 |  |
| Fulton |  |  |  |  |  |  |  |  |  |  |
| *Escherichia coli* | 31 | 74 | 100 | 97 | 94 | 100 | 93 | 93 | 87 |  |
| Greene |  |  |  |  |  |  |  |  |  |  |
| *Escherichia coli* | 118 | 63 | 98 | 98 | 88 | 98 | 88 | 93 | 84 |  |
| *Kings* |  |  |  |  |  |  |  |  |  |  |
| *Citrobacter diversus* | 89 |  | 97 |  | 99 | 89 | 99 | 100 | 99 |  |
| *Citrobacter freundii* | 43 |  | 88 |  | 93 | 93 | 93 | 98 | 88 |  |
| *Enterobacter aerogenes* | 106 |  | 92 |  | 100 | 24 | 99 | 99 | 98 |  |
| *Enterobacter cloacae* | 49 |  | 98 |  | 96 | 47 | 96 | 100 | 90 |  |
| Enterococcus Species | 1270 | 100 |  |  |  | 99 |  |  |  |  |
| *Escherichia coli* | 6463 | 49 | 92 | 88 | 74 | 97 | 73 | 89 | 71 |  |
| *Klebsiella pneumoniae* | 1098 |  | 95 | 93 | 95 | 40 | 95 | 96 | 89 |  |
| *Proteus mirabilis* | 408 | 82 | 99 | 93 | 86 |  | 86 | 94 | 85 |  |
| *Pseudomonas aeruginosa* | 184 |  | 95 |  | 74 |  | 68 | 98 |  |  |
| *Serratia marcescens* | 36 |  | 100 |  | 94 |  | 94 | 91 | 94 |  |
| *Staphylococcus aureus MRSA* | 64 |  |  |  | 14 | 100 | 16 |  | 98 | 86 |
| *Staphylococcus aureus MSSA* | 100 |  |  |  | 82 | 97 | 83 |  | 99 | 93 |

|  | n | Ampicillin | Ceftazidime | Cefazolin | Ciprofloxacin | Nitrofurantoin | Levofloxacin | Tobramycin | TMP/SMX | Tetracycline |
| --- | --- | --- | --- | --- | --- | --- | --- | --- | --- | --- |
| New York |  |  |  |  |  |  |  |  |  |  |
| *Citrobacter diversus* | 151 |  | 99 |  | 99 | 93 | 99 | 100 | 97 |  |
| *Citrobacter freundii* | 44 |  | 93 |  | 93 | 93 | 91 | 98 | 86 |  |
| *Enterobacter aerogenes* | 139 |  | 94 |  | 98 | 21 | 98 | 100 | 100 |  |
| *Enterobacter cloacae* | 59 |  | 90 |  | 90 | 42 | 90 | 88 | 73 |  |
| Enterococcus species | 1360 | 100 |  |  |  | 99 |  |  |  |  |
| *Escherichia coli* | 8147 | 52 | 92 | 90 | 79 | 97 | 79 | 90 | 72 |  |
| *Klebsiella pneumoniae* | 1215 |  | 94 | 91 | 94 | 43 | 94 | 95 | 88 |  |
| *Proteus mirabilis* | 464 | 79 | 100 | 94 | 94 |  | 94 | 96 | 91 |  |
| *Pseudomonas aeruginosa* | 116 |  | 93 |  | 82 |  | 75 | 95 |  |  |
| *Staphylococcus aureus MSSA* | 130 |  |  |  | 81 | 98 | 84 | 98 |  | 92 |
| Orange |  |  |  |  |  |  |  |  |  |  |
| Enterococcus Species | 175 | 97 |  |  |  | 99 |  |  |  |  |
| *Escherichia coli* | 1654 | 53 | 95 | 92 | 85 | 95 | 85 | 91 | 79 |  |
| *Klebsiella pneumoniae* | 202 |  | 100 | 99 | 100 | 47 | 100 | 100 | 95 |  |
| *Proteus mirabilis* | 65 | 80 | 100 | 89 | 89 |  | 89 | 97 | 91 |  |
| *Pseudomonas ­­aeruginosa* | 34 |  | 91 |  | 59 |  | 55 | 90 |  |  |
| Putnam |  |  |  |  |  |  |  |  |  |  |
| Enterococcus Species | 30 | 100 |  |  |  | 100 |  |  |  |  |
| *Escherichia coli* | 203 | 65 | 100 | 99 | 87 | 98 | 87 | 94 | 80 |  |
| Queens |  |  |  |  |  |  |  |  |  |  |
| *Citrobacter diversus* | 168 |  | 100 |  | 100 | 92 | 100 | 100 | 99 |  |
| *Citrobacter freundii* | 46 |  | 98 |  | 91 | 98 | 84 | 98 | 84 |  |
| *Enterobacter aerogenes* | 126 |  | 94 |  | 98 | 17 | 98 | 100 | 98 |  |
| *Enterobacter cloacae* | 47 |  | 86 |  | 98 | 43 | 98 | 98 | 91 |  |
| Enterococcus Species | 1659 | 100 |  |  |  | 99 |  |  |  |  |
| *Escherichia coli* | 8006 | 47 | 91 | 89 | 75 | 97 | 75 | 89 | 69 |  |
| *Klebsiella pneumoniae* | 1235 |  | 95 | 92 | 91 | 42 | 94 | 95 | 91 |  |
| *Proteus mirabilis* | 567 | 78 | 99 | 90 | 91 |  | 92 | 93 | 84 |  |
| *Pseudomonas aeruginosa* | 109 |  | 94 |  | 76 |  | 67 | 94 |  |  |
| *Serratia marcescens* | 61 |  | 100 |  | 100 |  | 98 | 89 | 100 |  |
| *Staphylococcus aureus MRSA* | 52 |  |  |  | 35 | 96 | 40 |  | 98 | 79 |
| *Staphylococcus aureus MSSA* | 140 |  |  |  | 87 | 99 | 88 |  | 100 | 85 |

|  | n | Ampicillin | Ceftazidime | Cefazolin | Ciprofloxacin | Nitrofurantoin | Levofloxacin | Tobramycin | TMP/SMX | Tetracycline |
| --- | --- | --- | --- | --- | --- | --- | --- | --- | --- | --- |
| Richmond |  |  |  |  |  |  |  |  |  |  |
| Enterococcus Species | 385 | 100 |  |  |  | 99 |  |  |  |  |
| *Escherichia coli* | 2347 | 52 | 92 | 89 | 79 | 97 | 79 | 90 | 76 |  |
| *Klebsiella pneumoniae* | 316 |  | 100 | 99 | 99 | 47 | 99 | 100 | 94 |  |
| *Proteus mirabilis* | 115 | 73 | 98 | 86 | 92 |  | 94 | 90 | 80 |  |
| *Pseudomonas aeruginosa* | 48 |  | 94 |  | 75 |  | 62 | 89 |  |  |
| Rockland |  |  |  |  |  |  |  |  |  |  |
| Enterococcus Species | 137 | 99 |  |  |  | 99 |  |  |  |  |
| *Escherichia coli* | 995 | 50 | 94 | 90 | 75 | 96 | 75 | 90 | 72 |  |
| *Klebsiella pneumoniae* | 118 |  | 99 | 98 | 98 | 42 | 97 | 99 | 90 |  |
| *Proteus mirabilis* | 58 | 78 | 98 | 91 | 86 |  | 86 | 95 | 86 |  |
| *Pseudomonas aeruginosa* | 35 |  | 97 |  | 74 |  | 71 | 94 |  |  |
| Suffolk |  |  |  |  |  |  |  |  |  |  |
| *Citrobacter diversus* | 53 |  | 100 |  | 98 | 91 | 98 | 100 | 100 |  |
| *Citrobacter freundii* | 46 |  | 80 |  | 96 | 98 | 91 | 98 | 78 |  |
| *Enterobacter aerogenes* | 82 |  | 91 |  | 99 | 13 | 99 | 100 | 100 |  |
| *Enterobacter cloacae* | 51 |  | 90 |  | 98 | 41 | 98 | 98 | 88 |  |
| Enterococcus Species | 858 | 100 |  |  |  | 98 |  |  |  |  |
| *Escherichia coli* | 5508 | 54 | 93 | 90 | 77 | 98 | 77 | 91 | 75 |  |
| *Klebsiella pneumoniae* | 860 |  | 97 | 96 | 98 | 41 | 97 | 97 | 91 |  |
| *Proteus mirabilis* | 316 | 78 | 99 | 95 | 86 |  | 88 | 97 | 85 |  |
| *Pseudomonas aeruginosa* | 115 |  | 96 |  | 73 |  | 62 | 97 |  |  |
| *Serratia marcescens* | 33 |  | 100 |  | 94 |  | 97 | 91 | 97 |  |
| *Staphylococcus aureus MSSA* | 79 |  |  |  | 85 | 100 | 84 |  | 100 | 95 |
| Sullivan |  |  |  |  |  |  |  |  |  |  |
| *Escherichia coli* | 85 | 44 | 98 | 95 | 93 | 98 | 93 | 92 | 79 |  |
| Ulster |  |  |  |  |  |  |  |  |  |  |
| *Escherichia coli* | 154 | 66 | 99 | 94 | 88 | 98 | 88 | 95 | 84 |  |

|  | n | Ampicillin | Ceftazidime | Cefazolin | Ciprofloxacin | Nitrofurantoin | Levofloxacin | Tobramycin | TMP/SMX | Tetracycline | Penicillin |
| --- | --- | --- | --- | --- | --- | --- | --- | --- | --- | --- | --- |
| Westchester |  |  |  |  |  |  |  |  |  |  |  |
| *Citrobacter diversus* | 111 |  | 100 |  | 99 | 84 | 99 | 99 | 99 |  |  |
| *Citrobacter freundii* | 58 |  | 93 |  | 95 | 97 | 93 | 98 | 92 |  |  |
| *Enterobacter aerogenes* | 92 |  | 91 |  | 100 | 20 | 100 | 100 | 99 |  |  |
| *Enterobacter cloacae* | 45 |  | 96 |  | 89 | 44 | 89 | 98 | 81 |  |  |
| Enterococcus Species | 1247 | 100 |  |  |  | 99 |  |  |  |  | 100 |
| *Escherichia coli* | 6703 | 54 | 93 | 91 | 81 | 98 | 81 | 92 | 76 |  |  |
| *Klebsiella pneumoniae* | 996 |  | 95 | 94 | 96 | 40 | 96 | 97 | 91 |  |  |
| *Proteus mirabilis* | 423 | 82 | 99 | 91 | 90 |  | 91 | 97 | 89 |  |  |
| *Pseudomonas aeruginosa* | 218 |  | 94 |  | 75 |  | 65 | 94 |  |  |  |
| *Serratia marcescens* | 37 |  | 100 |  | 95 |  | 95 | 84 | 95 |  |  |
| *Staphylococcus aureus MRSA* | 54 |  |  |  | 19 | 100 | 24 |  | 94 | 83 |  |
| *Staphylococcus aureus MSSA* | 122 |  |  |  | 85 | 98 | 87 |  | 100 | 91 |  |
