# Supplementary material for: Antimicrobial Susceptibility Trends Observed in Urinary Pathogens Obtained From New York State
Source: Open Forum Infect Dis. 2018 Nov 16;5(11):ofy297. doi: 10.1093/ofid/ofy297 (PMC6284462; doi:10.1093/ofid/ofy297)
Supplement: Supplemental Table 2 [file ofy297_suppl_supplemental_table_2.docx]

Supplemental Table 2. Antimicrobial Resistance in the Top 5 Isolates Compared By Age Group

|  | Age Group | | |  |
| --- | --- | --- | --- | --- |
| Nitrofurantoin | 10-20 | 15-49 | 18-30 | P value |
| *E. coli* | 98.2 | 98.1 | 98.1 | 0.92 |
| *Enterococcus* | 99.2 | 99.2 | 99.4 | 0.83 |
| *K. pneumoniae* | 57.5 | 60.8 | 57.5 | 0.14 |
| *Proteus spp* | N/A | N/A | N/A | N/A |
| *P. aeruginosa* | N/A | N/A | N/A | N/A |
| Trimethoprim-Sulfamethoxazole |  |  |  |  |
| *E. coli* | 78.2 | 76.0 | 76.5 | 0.02 |
| *Enterococcus* | N/A | N/A | N/A | N/A |
| *K. pneumoniae* | 89.4 | 91.8 | 92.4 | 0.22 |
| *Proteus spp* | 87.4 | 91.5 | 91.6 | 0.18 |
| *P. aeruginosa* | N/A | 85.4 | N/A | N/A |
| Ciprofloxacin |  |  |  |  |
| *E. coli* | 92.5 | 87.6 | 89.4 | < 0.01 |
| *Enterococcus* |  |  |  |  |
| *K. pneumoniae* | 98.5 | 97.3 | 97.5 | 0.45 |
| *Proteus spp* | 95.8 | 96.7 | 97.6 | 0.69 |
| *P. aeruginosa* | N/A | N/A | N/A | N/A |

Note: P-values are for chi-squared test for 3 x 2 tables.
