# Supplementary figures and images for: Antimicrobial Susceptibility Trends Observed in Urinary Pathogens Obtained From New York State
Source: Open Forum Infect Dis. 2018 Nov 16;5(11):ofy297. doi: 10.1093/ofid/ofy297 (PMC6284462; doi:10.1093/ofid/ofy297)

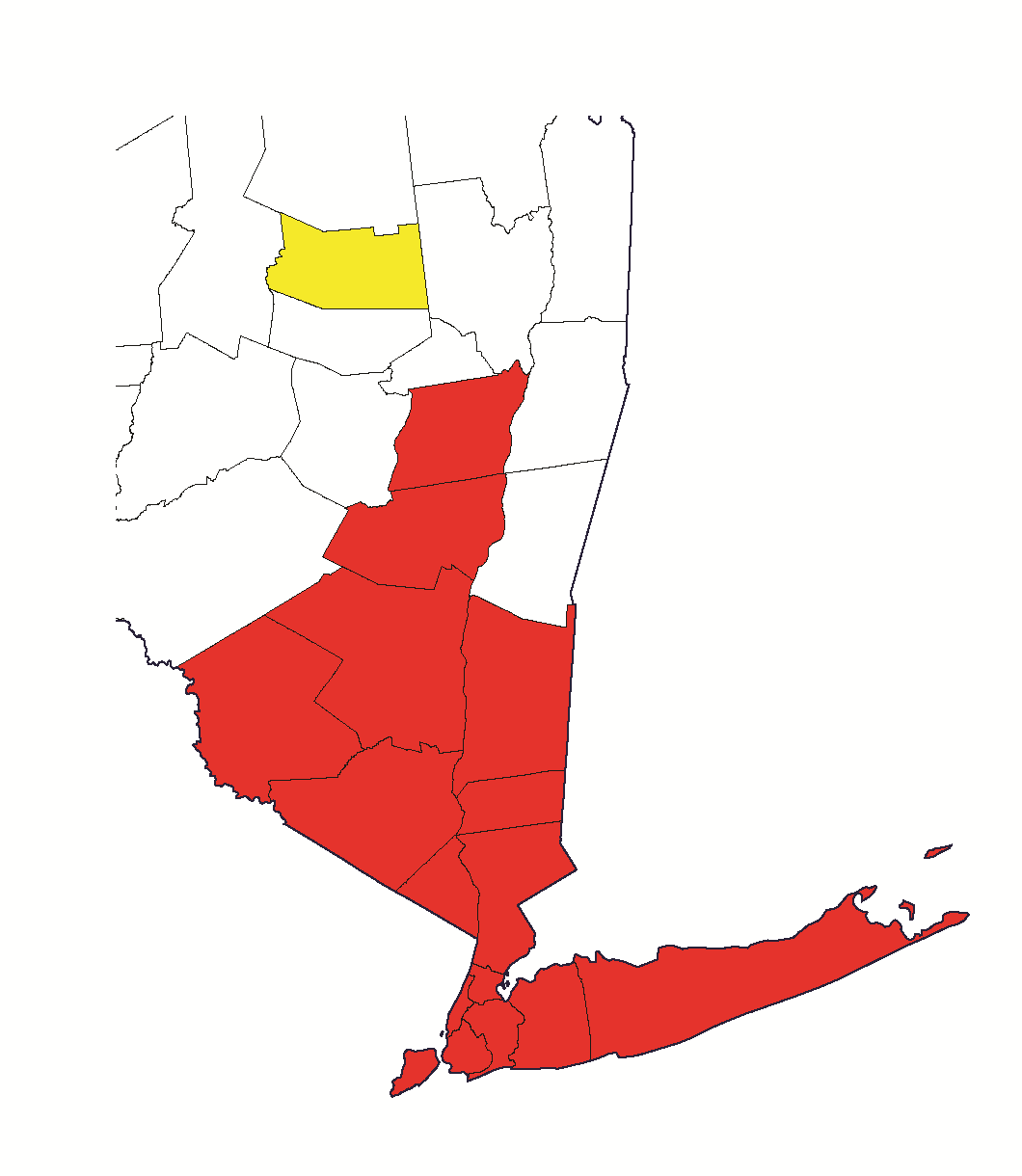

Supplement: Supplemental Figure 1 [file ofy297_suppl_supplemental_figure_1.png]

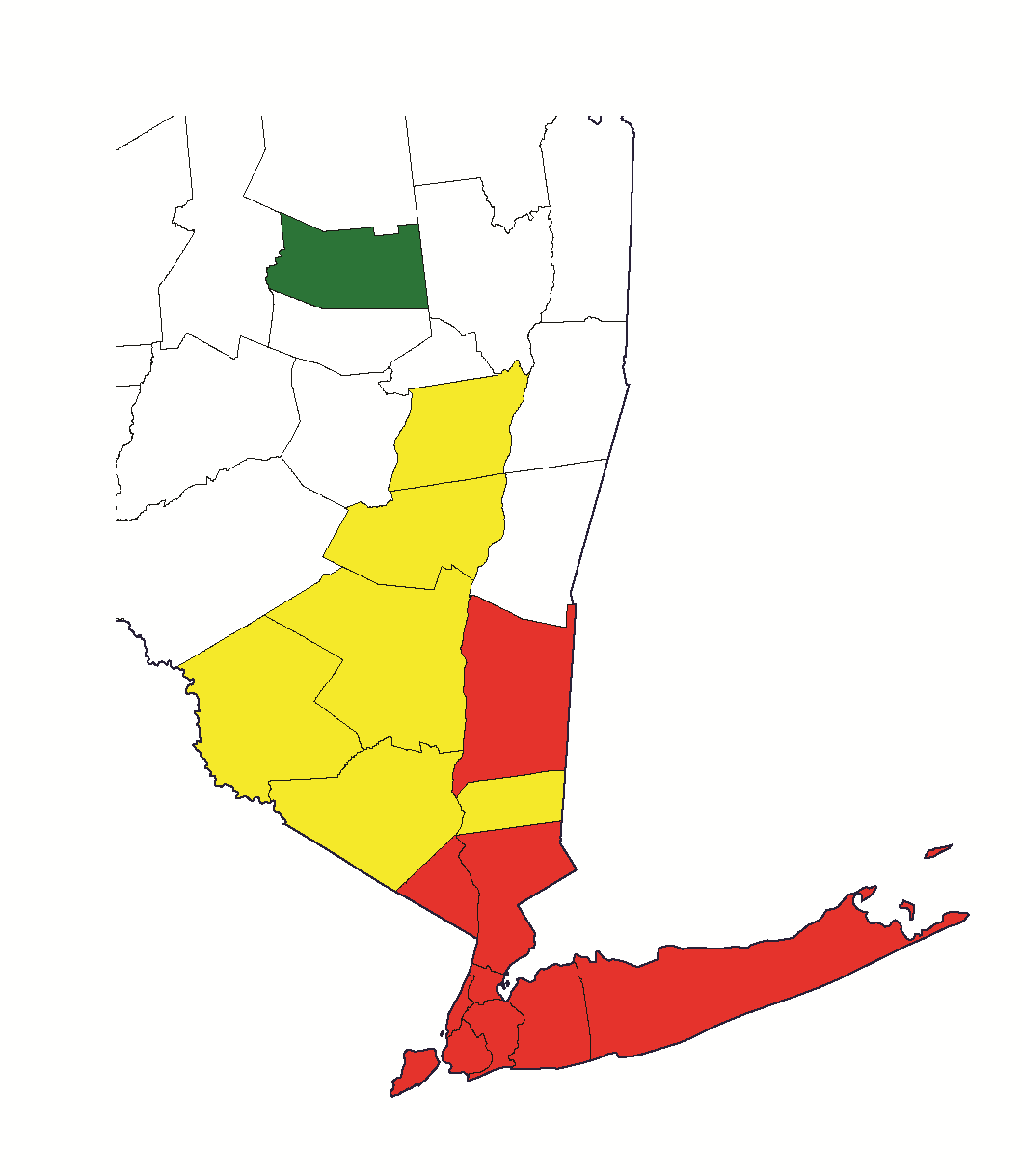

Supplement: Supplemental Figure 2 [file ofy297_suppl_supplemental_figure_2.png]

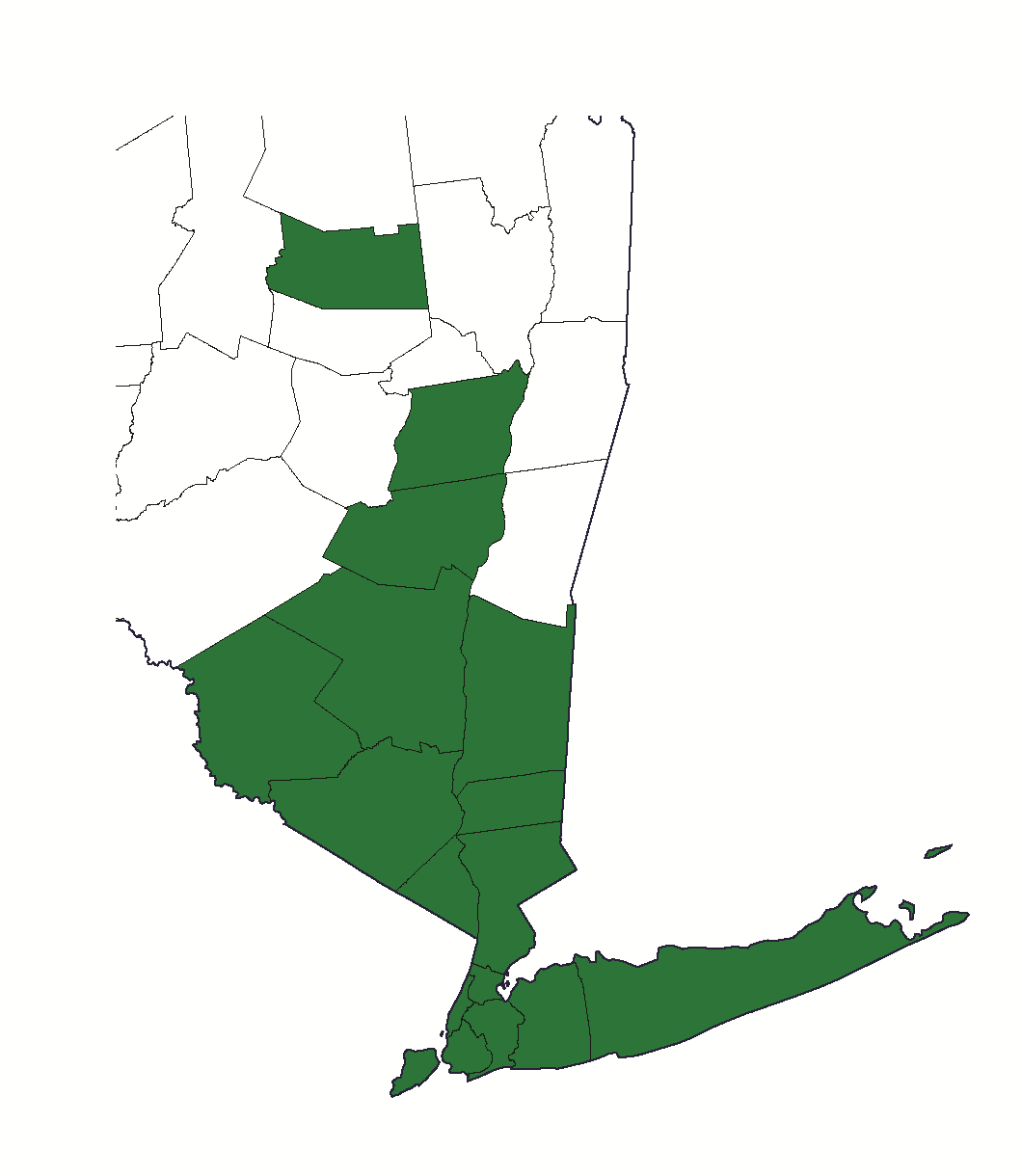

Supplement: Supplemental Figure 3 [file ofy297_suppl_supplemental_figure_3.png]
